# Risk of needing completion thyroidectomy for low‐risk papillary thyroid cancers treated by lobectomy

**DOI:** 10.1002/bjs5.50137

**Published:** 2019-02-06

**Authors:** A. N. DiMarco, M. S. Wong, J. Jayasekara, D. Cole‐Clark, A. Aniss, A. R. Glover, L. W. Delbridge, M. S. Sywak, S. B. Sidhu

**Affiliations:** ^1^ Endocrine Surgery Unit, Faculty of Health Sciences University of Sydney Sydney New South Wales Australia; ^2^ Department of Surgery and Cancer Imperial College London London UK

## Abstract

**Background:**

Low‐risk differentiated thyroid cancers may, according to the American Thyroid Association (ATA) 2015 guidelines, be managed initially with lobectomy. However, definitive risk categorization requires pathological assessment of the specimen, resulting in completion thyroidectomy being recommended when discordance between preoperative and postoperative staging occurs. This study sought to establish the expected rate of completion thyroidectomy in patients with papillary thyroid cancer (PTC) treated by lobectomy.

**Methods:**

Patients with PTC treated over 5 years (2013–2017 inclusive) and meeting the ATA criteria for lobectomy were identified from the prospectively developed database of a high‐volume, university department of endocrine surgery. Concordance between the ATA initial and final recommendation, and the putative rate of completion thyroidectomy were calculated. Multivariable analysis was used to assess preoperative factors as predictors of the need for total thyroidectomy.

**Results:**

Of 275 patients with PTC who met ATA preoperative criteria for lobectomy there was concordance between this and the final recommendation in 158 (57·5 per cent) and discordance in 117 (43·5 per cent). Most common reasons for discordance were: angioinvasion (30·8 per cent), local invasion (23·9 per cent) or both (20·5 per cent). Four patients (1·5 per cent) had permanent hypoparathyroidism. On multivariable analysis, age, sex, tumour size and family history did not independently predict the final treatment required.

**Conclusion:**

Although many patients may be treated adequately with lobectomy, just under half would require completion thyroidectomy. Further work is needed on preoperative risk stratification but, before this, total thyroidectomy remains the treatment of choice for low‐risk 1–4‐cm PTC in the hands of high‐volume thyroid surgeons who can demonstrate low complication rates.

## Introduction

The incidence of differentiated thyroid cancers (DTCs) has increased in recent decades^1^. A trend towards more conservative surgical treatment has gained traction, driven by low mortality associated with this condition, combined with concern about complication rates and the desire to avoid lifelong treatment with thyroxine^2^, ^3^. There are, however, conflicting views on the ideal extent of surgery and its effect on long‐term outcomes^4^, ^5^.

The American Thyroid Association (ATA) guidelines of 2015^2^ recommend thyroid lobectomy as a viable treatment option in low‐risk DTC, representing a significant shift away from the 2009 ATA guideline^6^. Low‐risk DTCs are defined as being less than 4 cm in size with no evidence of angioinvasion, local invasion or lymph node involvement. Thyroid lobectomies were previously recommended only for small (less than 1 cm) unifocal lesions, and total thyroidectomy for all lesions of 1 cm or larger. Although lesion size and the presence of lymph node metastases can be assessed before surgery, other features that are indications for total thyroidectomy, such as angioinvasion and local invasion, are apparent only at histopathological analysis of the surgical specimen. Completion thyroidectomy is therefore necessary in the subset of patients undergoing lobectomy for preoperative ‘low‐risk’ DTC that is then found to contain other features on histology.

Proponents of more conservative surgical approaches cite the need to account for differing complication rates from surgeons of all skills sets as reasons to change guidelines^2^, ^3^, ^7^. This swing away from potential overtreatment, in a bid to avoid complications from surgery that is not necessarily life‐prolonging, may now be resulting in the reverse, leading to undertreatment of DTC initially labelled as low risk^8^.

PTC comprises 85 per cent of all DTCs and is associated with the best prognosis for all thyroid cancer subtypes^2^, ^9^. Although other authors^8^ have examined the rate of completion procedures for DTC in general, the rate of completion procedures for PTC has not been investigated specifically. This study therefore sought to assess the rate of lobectomy and subsequent need for completion thyroidectomy for PTC, based on the 2015 ATA guidelines, in a cohort of patients treated in a high‐volume, university department of endocrine surgery.

## Methods

Following appropriate ethical review board approval, data were drawn from the prospectively developed database of consecutive patients undergoing surgery in a university teaching hospital department of endocrine surgery performing over 1500 procedures (thyroid, parathyroid and adrenal) per annum. All patients with PTC whose preoperative risk factors would have rendered them suitable for lobectomy according to the 2015 ATA guidelines 35(B)^2^ over a 5‐year interval (from January 2013 to December 2017 inclusive) were included. Demographic data, type of surgery performed, any postoperative complications and final histopathology were analysed.

Patients were diagnosed using fine‐needle aspiration biopsy, and staged by an endocrine surgeon based on clinical examination and imaging with one or more of ultrasonography, CT or MRI. Routine prophylactic ipsilateral central compartment dissection (pCND) was performed in all patients, based on the potential for reduction in the rate of local recurrence and avoidance of reoperation as set against a low morbidity for the procedure in this unit^10^, ^11^. The presence of *BRAF* V600E mutation or microscopic extrathyroidal extension was not considered an indication for total thyroidectomy.

All patients in the database with a final diagnosis of PTC were included, and suitable patients selected using the following exclusion criteria: tumour size less than 1 or greater than 4 cm, invasion into surrounding structures, clinical or radiological lymph node metastases, and Bethesda I–IV aspiration biopsy result.

### Outcomes

The primary outcome measure was concordance between the ATA 2015 initial recommendation for lobectomy and the final recommendation based on histopathology: no further surgery or completion thyroidectomy. The presence of histological features leading to an intermediate‐risk classification according to the ATA 2015 guidelines^2^ would usually attract a recommendation for treatment with radioactive iodine (RAI) at this institution and therefore requires completion thyroidectomy to facilitate this. These intermediate‐risk features are as listed in Table 11 of the ATA guidelines: angioinvasion, local invasion (minimal extension into perithyroidal soft tissue or sternohyoid muscle), five or more positive central compartment lymph nodes or positive lateral compartment lymph nodes.

A secondary analysis was performed to establish whether any preoperative factor was able to predict the requirement for completion thyroidectomy. Preoperative factors assessed were age, sex, family history and tumour size.

### Statistical analysis

The demographics and primary outcome measure of concordance between the ATA 2015 initial and final treatment recommendation were expressed using simple descriptive statistics: median (range), and percentage concordance *versus* rate of completion thyroidectomy. The secondary analysis was performed using simple bivariable correlation and multivariable logistic regression. All statistics were calculated in SPSS® version 24 (IBM, Armonk, New York, USA).

## Results

**Figure 1:**
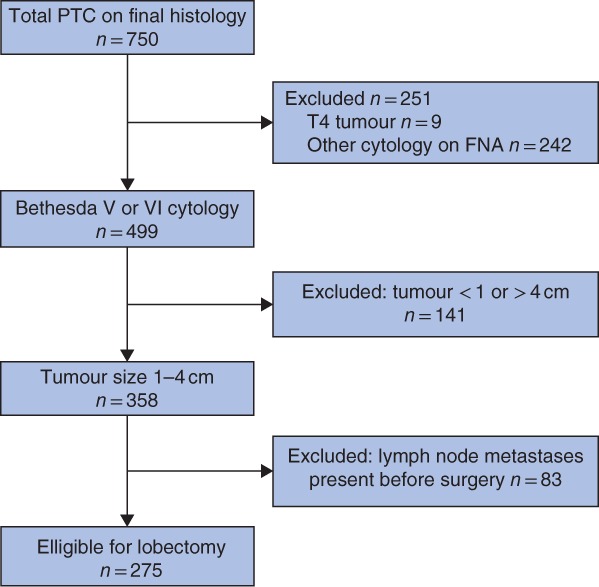
Flow chart showing patient selection. PTC, papillary thyroid cancer; FNA, fine‐needle aspiration

Some 750 patients who underwent thyroidectomy between 1 January 2013 and 31 December 2017 were diagnosed with PTC as the primary pathology on histology. *Fig*. 1 shows the selection of patients who would have been suitable for lobectomy according to the 2015 ATA guidelines. Of these, nine were excluded owing to gross invasion based on preoperative imaging or perioperative findings. Of the remaining 741 patients, 499 (67·3 per cent) had a preoperative aspiration biopsy result of Bethesda V or VI and 358 (47·7 per cent of all PTCs) were also between 1 and 4 cm in size. Preoperative evaluation identified 83 patients who had lymph node metastases present on clinical examination or imaging, leaving 275 whose tumours would have been classified as low risk and been eligible for lobectomy as the initial management, representing 36·7 per cent of all PTCs.

Median age in this low‐risk group was 51 (range 19–84) years and 228 patients (82·9 per cent) were women. Median tumour size was 15 (range 10–35) mm. Total thyroidectomy and central compartment lymph node dissection was performed in 253 patients (92·0 per cent). Patients were counselled routinely about the option of undergoing hemithyroidectomy and the possibility of further completion surgery being required. Some 8·0 per cent chose to undergo hemithyroidectomy. Prophylactic central lymph node dissection was also performed in these patients.

**Figure 2:**
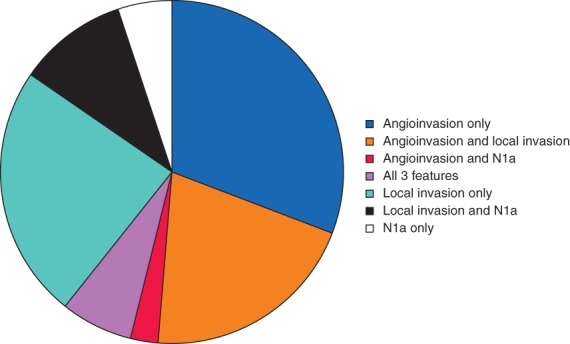
Features leading to recommendation for completion thyroidectomy

Analysis of the final histopathology of these 275 patients showed concordance between the initial ATA recommendation for lobectomy and final recommendation in 158 (57·5 per cent), but discordance, representing a requirement for completion thyroidectomy, in 117 (42·5 per cent). Features that would have attracted the recommendation for completion thyroidectomy are shown in *Fig*. 2. These were angioinvasion alone in 36 of 117 patients (30·8 per cent), local invasion alone in 28 (23·9 per cent), greater than five involved central lymph nodes in six (5·1 per cent) and multiple factors in 47 (40·2 per cent).

Complication rates were low. A single patient developed a haematoma requiring surgical evacuation, there were eight temporary palsies of the recurrent laryngeal nerve (2·9 per cent) and two recurrent laryngeal nerves were sacrificed owing to involvement with advanced tumours (both T3 N1a). There were no inadvertent permanent recurrent laryngeal nerve injuries. All patients were managed with prophylactic oral calcium in the immediate postoperative period, with four developing permanent hypoparathyroidism (1·5 per cent).

There were three local recurrences in the cohort. These included two patients who would have been stratified as low risk and potentially offered hemithyroidectomy according to the ATA guidelines, and one stratified as intermediate risk. One of the low‐risk patients underwent hemithyroidectomy as the initial surgery and subsequently required completion thyroidectomy.

**Figure 3:**
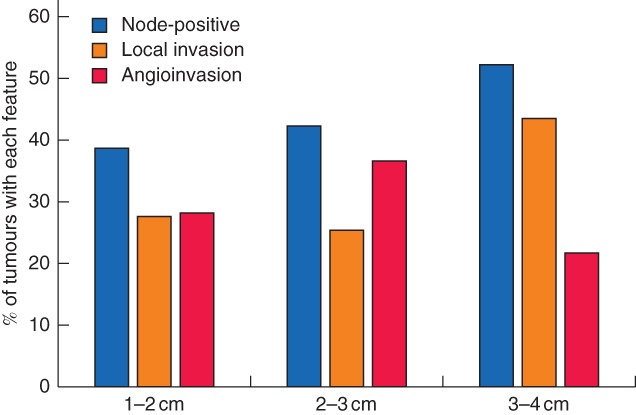
Factors favouring completion thyroidectomy by tumour size

*Fig*. 3 shows the incidence of features leading to a recommendation for completion thyroidectomy by increasing tumour size. Simple bivariable analysis of the association between age, sex, tumour size, family history and the aspiration biopsy result (whether Bethesda V or VI) and the final recommendation showed that patient age had the greatest correlation with recommended outcome (*R* = 0·105), but this was not statistically significant (*P* = 0·081). No statistically significant correlation was found between the final recommendation and any other variable. All variables were then entered into a multivariable logistic regression model; again, age came the closest to statistical significance (B = 0·015, *P* = 0·088) but no statistically significant independent predictor of the need for completion thyroidectomy was found.

## Discussion

The change in guidelines away from total thyroidectomy to lobectomy has been a topic of much debate for several reasons. Interpretation of the data that led to the change has been questioned. Emphasis on survival as the primary outcome measure neglects the issue of local recurrence; this is arguably of greater importance in lower‐risk cancers as there is concern that, in a bid to avoid potential overtreatment, the reverse will occur. Even if survival is not significantly affected, an increase in local recurrence may be seen in the coming years. The logic of avoiding complications by reducing the extent of surgery loses sight of the fact that complication rates differ between low‐ and high‐volume surgeons. Finally, reoperation for completion thyroidectomy in the short term and reintervention for local recurrence in the longer term may nullify any benefit from avoidance of immediate complications.

The change in ATA guidelines was based on studies^12^, ^13^ that used multivariable analysis from large population studies showing no survival benefit from total thyroidectomy over lobectomy. This contradicted earlier studies using the same data^4^, highlighting the lack of consensus on the appropriate surgical management for this group. Studies arguing against total thyroidectomy have not included data on local recurrence, and an inherent limitation in large database studies is selection bias for either procedure. Considering the indolent nature of DTC, the impact of these changes may not be apparent for some time yet^14^.

Proponents of lobectomy cite the avoidance of serious complications (recurrent laryngeal nerve injury, hypoparathyroidism) and obviation of the need for lifelong thyroxine as justification for this approach^3^. A reduction in the occurrence of such complications in lobectomy compared with total thyroidectomy has been demonstrated for both low‐ and high‐volume thyroid surgeons, although the risks are significantly lower when the procedure is performed by a high‐volume surgeon^15^, ^16^. In this study, although 92·0 per cent of patients had actually undergone total thyroidectomy with routine pCND, permanent complications affected only 1·5 per cent. Conversely, among those going on to require completion thyroidectomy, the risks conferred by two hospital admissions^17^, ^18^, two general anaesthetics^19^, and the associated psychological stress to the patient and family should not be neglected. The likelihood of completion thyroidectomy ultimately being recommended is therefore of paramount importance when discussing the optimal initial treatment option with patients. The argument for avoidance of thyroxine is not supported by patient‐reported quality‐of‐life studies^20^, and the fact that the lifetime prevalence of hypothyroidism requiring thyroxine is around 5 per cent and is increased anyway in those undergoing lobectomy^21^.

The advocacy for total thyroidectomy is linked to administration of iodine‐131, facilitation of monitoring with serum thyroglobulin (TG) and analysis of the contralateral thyroid lobe for other foci of disease. Although the benefit of iodine‐131, whether for remnant ablation or cancer therapy, has been shown in an unselected retrospectively analysed cohort^22^, subsequent assessment of the benefit in patients with low‐risk cancers has not been proven^23^, ^24^. However, it is also unclear for the intermediate‐risk groups^25^, ^26^. Data on RAI administration in this study cohort were not complete; however, the overall rate over the study interval for 1–4‐cm PTCs was 36·3 per cent (272 of 750 patients). Further evidence regarding the efficacy of RAI remnant ablation in low‐ and intermediate‐risk PTC, and changes in indications for postoperative RAI may provide a better justification for lobectomy *versus* total thyroidectomy in the future.

The use of serial serum TG measurement for post‐treatment monitoring has been debated. Although pCND does not necessarily increase the rate of athyroglobulinaemia, TG is certainly not a meaningful marker in the presence of a remnant lobe^6^, ^10^, ^11^, ^27^. The cost of serial ultrasound imaging after lobectomy may exceed that of total thyroidectomy and serum TG follow‐up. Two studies^14^, ^28^ using predictive modelling both concluded that total thyroidectomy, even in low‐risk disease, was superior. It is accepted that these models will be affected by the frequency of imaging; the optimal interval as yet remains unknown.

Analysis of the contralateral lobe for occult carcinoma is another potential benefit of total thyroidectomy. Rates as high as 47 per cent have been reported^29^ and are of relevance, given that bilateral PTC has consistently been shown to be associated with a more aggressive course than unilateral disease^30^, ^31^.

The rate of completion thyroidectomy in this study was over 40 per cent, more than twice the 19·5 per cent rate in the cohort study of all DTCs^8^. Reasons for this may be exclusion of incidental PTCs (only those with preoperative cytology results of Bethesda V–VI were included), and the use of routine ipsilateral central compartment clearance, increasing the pick‐up rate of N1a disease which would favour 131‐iodine RAI and therefore completion thyroidectomy. Angioinvasion was seen in 25·8 per cent of patients in this cohort and only 12 per cent in that of Kluijfhout and colleagues^8^. The exact reasons for this difference are not known, but may relate to exclusion of follicular thyroid cancer and incidental PTC.

Variability in the expected rate of completion thyroidectomy and complications specific to an individual unit and surgeon highlight the difficulties in creating guidelines to cater for DTC managed in diverse settings. It is admirable that the ATA has sought to protect patients from the complications of unnecessary operations; however, using guidelines to encourage the low‐volume surgeon to perform a lower‐morbidity operation for DTC is only one way to achieve this. This study highlights the importance of surgeons understanding the characteristics of their own patient cohort, their individual complication rate and patient attitudes towards completion thyroidectomy, along with complications in each scenario and implications of lobectomy or surveillance, when selecting the most appropriate surgical option in low‐risk patients.

In the future, preoperative molecular analysis may assist in more accurate preoperative risk stratification and decision‐making but, in the meantime, guidelines such as those provided by the ATA should be applied flexibly to reflect differing circumstances of providers and patients. Further work on decision analysis based on the relative merits of the two approaches and patient preference may also be of benefit^14^, ^28^.

## References

[1] Australian Institute of Health and Welfare (AIHW) . Burden of Cancer in Australia: Australian Burden of Disease Study 2011. *Australian Burden of Disease Study series no. 12*. AIHW: Canberra, 2017.

[2] Haugen BR , Alexander EK , Bible KC , Doherty GM , Mandel SJ , Nikiforov YE *et al* 2015 American Thyroid Association management guidelines for adult patients with thyroid nodules and differentiated thyroid cancer: the American Thyroid Association guidelines task force on thyroid nodules and differentiated thyroid cancer. Thyroid 2016; 26: 1–133. 26462967 10.1089/thy.2015.0020 PMC4739132

[3] Welch HG , Doherty GM . Saving thyroids – overtreatment of small papillary cancers. N Engl J Med 2018; 379: 310–312. 30044933 10.1056/NEJMp1804426

[4] Barney BM , Hitchcock YJ , Sharma P , Shrieve DC , Tward JD . Overall and cause‐specific survival for patients undergoing lobectomy, near‐total, or total thyroidectomy for differentiated thyroid cancer. Head Neck 2011; 33: 645–649. 20687168 10.1002/hed.21504

[5] Ito Y , Miyauchi A , Kihara M , Fukushima M , Higashiyama T , Miya A . Overall survival of papillary thyroid carcinoma patients: a single‐institution long‐term follow‐up of 5897 patients. World J Surg 2018; 42: 615–622. 29349484 10.1007/s00268-018-4479-z PMC5801380

[6] American Thyroid Association (ATA) Guidelines Taskforce on Thyroid Nodules and Differentiated Thyroid Cancer , Cooper DS , Doherty GM , Haugen BR , Kloos RT , Lee SL *et al* Revised American Thyroid Association management guidelines for patients with thyroid nodules and differentiated thyroid cancer. Thyroid 2009; 19: 1167–1214. 19860577 10.1089/thy.2009.0110

[7] Takami H , Ito Y , Okamoto T , Yoshida A . Therapeutic strategy for differentiated thyroid carcinoma in Japan based on a newly established guideline managed by Japanese Society of Thyroid Surgeons and Japanese Association of Endocrine Surgeons. World J Surg 2011; 35: 111–121. 21042913 10.1007/s00268-010-0832-6

[8] Kluijfhout WP , Pasternak JD , Drake FT , Beninato T , Shen WT , Gosnell JE *et al* Application of the new American Thyroid Association guidelines leads to a substantial rate of completion total thyroidectomy to enable adjuvant radioactive iodine. Surgery 2017; 161: 127–133. 27855968 10.1016/j.surg.2016.05.056

[9] Aschebrook‐Kilfoy B , Ward MH , Sabra MM , Devesa SS . Thyroid cancer incidence patterns in the United States by histologic type, 1992–2006. Thyroid 2011; 21: 125–134. 21186939 10.1089/thy.2010.0021 PMC3025182

[10] Glover AR , Gundara JS , Norlén O , Lee JC , Sidhu SB . The pros and cons of prophylactic central neck dissection in papillary thyroid carcinoma. Gland Surg 2013; 2: 196–205. 25083483 10.3978/j.issn.2227-684X.2013.10.05 PMC4115751

[11] Popadich A , Levin O , Lee JC , Smooke‐Praw S , Ro K , Fazel M *et al* A multicenter cohort study of total thyroidectomy and routine central lymph node dissection for cN0 papillary thyroid cancer. Surgery 2011; 150: 1048–1057. 22136820 10.1016/j.surg.2011.09.003

[12] Mendelsohn AH , Elashoff DA , Abemayor E , St John MA . Surgery for papillary thyroid carcinoma: is lobectomy enough? Arch Otolaryngol Head Neck Surg 2010; 136: 1055–1061. 21079156 10.1001/archoto.2010.181

[13] Adam MA , Pura J , Gu L , Dinan MA , Tyler DS , Reed SD *et al* Extent of surgery for papillary thyroid cancer is not associated with survival: an analysis of 61 775 patients. Ann Surg 2014; 260: 601–607. 25203876 10.1097/SLA.0000000000000925 PMC4532384

[14] Shrime MG , Goldstein DP , Seaberg RM , Sawka AM , Rotstein L , Freeman JL *et al* Cost‐effective management of low‐risk papillary thyroid carcinoma. Arch Otolaryngol Head Neck Surg 2007; 133: 1245–1253. 18086967 10.1001/archotol.133.12.1245

[15] Sosa JA , Bowman HM , Tielsch JM , Powe NR , Gordon TA , Udelsman R . The importance of surgeon experience for clinical and economic outcomes from thyroidectomy. Ann Surg 1998; 228: 320–330. 9742915 10.1097/00000658-199809000-00005 PMC1191485

[16] Hauch A , Al‐Qurayshi Z , Randolph G , Kandil E . Total thyroidectomy is associated with increased risk of complications for low‐ and high‐volume surgeons. Ann Surg Oncol 2014; 21: 3844–3852. 24943236 10.1245/s10434-014-3846-8

[17] Leape LL . Errors in medicine. Clin Chim Acta 2009; 404: 2–5. 19302989 10.1016/j.cca.2009.03.020

[18] de Vries EN , Ramrattan MA , Smorenburg SM , Gouma DJ , Boermeester MA . The incidence and nature of in‐hospital adverse events: a systematic review. Qual Saf Health Care 2008; 17: 216–223. 18519629 10.1136/qshc.2007.023622 PMC2569153

[19] Newland MC , Ellis SJ , Lydiatt CA , Peters KR , Tinker JH , Romberger DJ *et al* Anesthetic‐related cardiac arrest and its mortality: a report covering 72 959 anesthetics over 10 years from a US teaching hospital. Anesthesiology 2002; 97: 108–115. 12131111 10.1097/00000542-200207000-00016

[20] Petersen K , Bengtsson C , Lapidus L , Lindstedt G , Nyström E . Morbidity, mortality, and quality of life for patients treated with levothyroxine. Arch Intern Med 1990; 150: 2077–2081. 2222093

[21] Chaker L , Bianco AC , Jonklaas J , Peeters RP . Hypothyroidism. Lancet 2017; 390: 1550–1562. 28336049 10.1016/S0140-6736(17)30703-1 PMC6619426

[22] Mazzaferri EL , Jhiang SM . Long‐term impact of initial surgical and medical therapy on papillary and follicular thyroid cancer. Am J Med 1994; 97: 418–428. 7977430 10.1016/0002-9343(94)90321-2

[23] DeGroot LJ , Kaplan EL , McCormick M , Straus FH . Natural history, treatment, and course of papillary thyroid carcinoma. J Clin Endocrinol Metab 1990; 71: 414–424. 2380337 10.1210/jcem-71-2-414

[24] Hay ID , Thompson GB , Grant CS , Bergstralh EJ , Dvorak CE , Gorman CA *et al* Papillary thyroid carcinoma managed at the Mayo Clinic during six decades (1940–1999): temporal trends in initial therapy and long‐term outcome in 2444 consecutively treated patients. World J Surg 2002; 26: 879–885. 12016468 10.1007/s00268-002-6612-1

[25] Sawka AM , Brierley JD , Tsang RW , Thabane L , Rotstein L , Gafni A *et al* An updated systematic review and commentary examining the effectiveness of radioactive iodine remnant ablation in well‐differentiated thyroid cancer. Endocrinol Metab Clin North Am 2008; 37: 457–480. 18502337 10.1016/j.ecl.2008.02.007

[26] Chan TW , Hurley JR , Fahey TJ . Postoperative radioactive iodine for differentiated thyroid cancer: a historical perspective. Clin Oncol 2017; 2: 1230.

[27] Barczyński M , Konturek A , Stopa M , Nowak W. Prophylactic central neck dissection for papillary thyroid cancer. Br J Surg 2013; 100: 410–418. 23188784 10.1002/bjs.8985

[28] Esnaola NF , Cantor SB , Sherman SI , Lee JE , Evans DB . Optimal treatment strategy in patients with papillary thyroid cancer: a decision analysis. Surgery 2001; 130: 921–930. 11742318 10.1067/msy.2001.118370

[29] Lv T , Zhu C , Di Z . Risk factors stratifying malignancy of nodules in contralateral thyroid lobe in patients with pre‐operative ultrasound indicated unilateral papillary thyroid carcinoma: a retrospective analysis from single centre. Clin Endocrinol (Oxf) 2018; 88: 279–284. 29083503 10.1111/cen.13506

[30] Wang W , Su X , He K , Wang Y , Wang H , Wang H *et al* Comparison of the clinicopathologic features and prognosis of bilateral *versus* unilateral multifocal papillary thyroid cancer: an updated study with more than 2000 consecutive patients. Cancer 2016; 122: 198–206. 26506214 10.1002/cncr.29689

[31] Qu N , Zhang L , Wu WL , Ji QH , Lu ZW , Zhu YX *et al* Bilaterality weighs more than unilateral multifocality in predicting prognosis in papillary thyroid cancer. Tumour Biol 2016; 37: 8783–8789. 26743781 10.1007/s13277-015-4533-5

